# Stable gene replacement in barley by targeted double-strand break induction

**DOI:** 10.1093/jxb/erv537

**Published:** 2015-12-27

**Authors:** Koichi Watanabe, Ulrike Breier, Götz Hensel, Jochen Kumlehn, Ingo Schubert, Bernd Reiss

**Affiliations:** ^1^Leibniz Institute of Plant Genetics and Crop Plant Research (IPK), D-06466 Gatersleben, Stadt Seeland, Germany; ^2^Max-Planck-Institute for Plant Breeding Research, Carl-von-Linne-Weg 10, D-50829 Cologne, Germany; ^3^Faculty of Science and Central European Institute of Technology, Masaryk University, 61137 Brno, Czech Republic

**Keywords:** Barley, double-strand break induction, gene replacement, gene targeting, homology-directed DNA integration, *Hordeum vulgare*, precision genome engineering.

## Abstract

Gene targeting is becoming an important tool for precision genome engineering in plants. During gene replacement, a variant of gene targeting, transformed DNA integrates into the genome by homologous recombination (HR) to replace resident sequences. We have analysed gene targeting in barley (*Hordeum vulgare*) using a model system based on double-strand break (DSB) induction by the meganuclease I-*Sce*I and a transgenic, artificial target locus. In the plants we obtained, the donor construct was inserted at the target locus by homology-directed DNA integration in at least two transformants obtained in a single experiment and was stably inherited as a single Mendelian trait. Both events were produced by one-sided integration. Our data suggest that gene replacement can be achieved in barley with a frequency suitable for routine application. The use of a codon-optimized nuclease and co-transfer of the nuclease gene together with the donor construct are probably the components important for efficient gene targeting. Such an approach, employing the recently developed synthetic nucleases/nickases that allow DSB induction at almost any sequence of a genome of interest, sets the stage for precision genome engineering as a routine tool even for important crops such as barley.

## Highlight

Using double-strand break induction in the crop plant barley, standard transformation technology allows gene replacement to occur at frequencies suitable for routine application.

## Introduction

The importance of gene targeting for gene function analysis and for production of transgenic organisms has increased enormously. The interest in gene targeting in plants was fuelled by new tools for targeted DNA double-strand break (DSB) induction that have become available lately (Belhaj *et al.*, 2013; Puchta and Fauser, 2013, 2014; Voytas, 2013; Osakabe and Osakabe, 2014). Their use in plants is just at the beginning, but zinc-finger nucleases (ZFNs) (Shukla *et al.*, 2009; Townsend *et al.*, 2009), transcription activator-like effector nucleases (TALENs) (Wood *et al.*, 2011; Qi *et al.*, 2013), or clustered regularly interspaced short palindromic repeats (CRISPR) and CRISPR-associated protein (Cas) (CRISPR–Cas) (Fauser *et al.*, 2014; Feng *et al.*, 2014) hold great promise for the future. At present these tools are primarily used for targeted mutagenesis; that is, the introduction of mutations at pre-determined positions in the genome by imprecise DSB repair via non-homologous end-joining (NHEJ). Reports of the use of such tools to optimize gene targeting in plants are still limited. Except for some rare reports (Shukla *et al.*, 2009; Townsend *et al.*, 2009; Ainley *et al.*, 2013) using direct DNA transfer for transformation, the efficiencies obtained are still low (Qi *et al.*, 2013). For targeted gene replacement in particular the efficiencies were not high enough for routine application in a setting suitable for commercial application. Transformation of almost all commercially important crops is entirely *Agrobacterium* mediated. The use of DSB induction in conjunction with *Agrobacterium*-mediated gene replacement turned out to be more challenging than targeted mutagenesis. The efficiencies reported thus far are highly variable. These were quite high in maize (*Zea mays*) or tobacco (*Nicotiana tabacum*) using the meganuclease I-*Sce*I (D’Halluin *et al.*, 2008) and a validated zinc finger nuclease (ZFN) (Cai *et al.*, 2009), or rather low in *Arabidopsis thaliana* using a different ZFN (De Pater *et al.*, 2009). These differences could reflect differences in the organisms, the efficiencies of the nucleases, or the experimental designs employed.

The monocotyledonous diploid seed plant barley (*Hordeum vulgare*) is an important cereal crop. Extended mutant collections and tilling (Targeting Induced Local Lesions in Genomes) populations (Lundqvist and Franckowiak, 2003; Gottwald *et al.*, 2009; Lundqvist, 2014) make this plant an attractive model for the more complex Triticeae such as wheat (Mayer *et al.*, 2011). Moreover, extensive genomic tools and reliable transformation protocols are available for barley. In particular, a draft sequence of the barley genome (IntBarleySeqCon, 2012) and the possibility to generate transgenic plants efficiently by *Agrobacterium*-mediated transformation (Tingay *et al.*, 1997) laid the foundation for reverse genetics approaches in barley, including genome editing and precision gene replacement.

To demonstrate the feasibility of targeted gene replacement in barley, we decided to use the meganuclease I-*Sce*I as a well characterized DSB induction tool. A transgenic gene-targeting assay system was established and used to determine efficiencies in the absence and presence of DSB induction. The results presented here show that multiple gene replacement events can be obtained in a single standard transformation experiment in barley using co-introduction of the nuclease gene together with the donor construct.

## Materials and methods

### Vector constructions

Plasmid UBIp-ABM (DNA Cloning Service e.K., Hamburg, Germany) is a vector carrying the promoter, the 5'-untranslated exon, and the first intron (*ubiquitin* promoter) of the maize *ubiquitin* (*Ubi-1*) gene (Christensen and Quail, 1996), multicloning sites, and the NOS terminator. The plasmid was modified to obtain the plasmid UBIp-ABM1256 by inserting an adaptor fragment carrying *Sfi*I and *Acc*65I recognition sites upstream of the *ubiquitin* promoter and an adaptor fragment carrying a *Hin*dIII recognition site downstream of the NOS terminator.

The recipient construct p6Um was obtained as follows: an I-*Sce*I recognition site harbouring a DNA fragment obtained by annealing of oligos Ada15 (5'-CAT GGA GGA GTA GGG ATA ACA GGG TAA TCA-3') and Ada16 (5'-TAT GAT TAC CCT GTT ATC CCT ACT CCTC-3') was inserted into the first *Nco*I and the *Nde*I recognition sites within the open reading frame of the *hpt* gene to obtain *hpt*Δ. Then *hpt*Δ was inserted into the binary vector p6U (DNA Cloning Service e.K.) in exchange for the intact *hpt* gene present in this plasmid. A DNA fragment containing the *pat* gene was extracted from the plasmid p7U (DNA Cloning Service e.K.) by digestion with *Mlu*I and *Xho*I, and the fragment was inserted into the plasmid UBIp-ABM1256, resulting in the plasmid PAT/UBIp-ABM1256. The plasmid PAT/UBIp-ABM1256 was cleaved with *Sfi*I and *Hin*dIII, and a 2.3kb DNA fragment carrying the *pat* gene driven by the *ubiquitin* promoter was cloned into the p6U plasmid harbouring the mutagenized hygromycin resistance gene to yield the final recipient construct p6Um.

The two donor constructs, one including the expressible I-*SceI* coding region, PAT-I-SceI/p6U-pro, and one without it, PAT/p6U-pro, were obtained from the intermediate plasmid p6U-pro. This was generated from plasmid p6U by replacing the 2.8kb *Xba*I and *Bam*HI segment carrying the *Ubi-1* promoter and the *hpt* gene with a 1.7kb *Nhe*I and *Bam*HI fragment from the same plasmid. This step eliminated the *Ubi-1* promoter in front of the *hpt* gene. The donor construct PAT/P6U-pro was obtained by inserting the *pat* gene from PAT/UBIp-ABM1256 as a *Sfi*I and *Hin*dIII fragment into the corresponding restriction sites of p6U-pro. The donor construct PAT-I-SceI/p6U-pro carrying the additional I-*SceI* gene was obtained by inserting the *Ubi-1* promoter-driven codon-optimized I-*Sce*I endonuclease gene together with the *Ubi-1* promoter-driven *pat* gene from the construct of Vu *et al.* (2014) into the multicloning site of p6U-pro.

### FISH analysis

For the preparation of chromosomes, seeds were soaked in water for 2 d at 4 °C and then placed on wet filter paper for 2 d at room temperature. To increase the number of mitotic chromosomes, seedlings were cultured at room temperature on filter paper soaked with 1.25mM hydroxyurea (Sigma-Aldrich Chemie GmbH, Taufkirchen, Germany) for 18h, with water for 5h, and subsequently with 4 µM amiprophos-methyl (Duchefa Biochemie B.V, Haarlem, The Netherlands) for 2h. Then seedlings were treated with ice-cold water overnight and fixed in ethanol/acetic acid (3:1) overnight at 4 °C. After washes in water and citrate buffer [10mM Na-citrate (pH 4.5)], root tips were digested in an enzyme mixture containing 2% cellulase Onozuka R-10 and 0.5% Pectolyase Y-23 in citrate buffer at 37 °C. The macerated plant tissue was suspended in acetic acid/methanol (3:1). A drop of the suspension was dropped on a glass slide to spread the metaphase cells.

To obtain probes for fluorescence *in situ* hybridization (FISH), DNA was labelled by nick translation as described (Jovtchev *et al.*, 2008). The DNA fragment of the 5S rDNA coding and flanking non-coding regions (GeneBank accession number S70723) was amplified by PCR with primers (Hv5S_For, 5'-GGA TGC GAT CAT ACC AGCAC-3'; Hv5S_Rev, 5'-GAC ATG CAA CTA TCT ATT TGT-3') and barley genomic DNA as template. The amplification product and the plasmid PAT/UBIp-ABM1256 were labelled with Alexa Fluor 488-5-dUTP and Texas Red-12-dUTP (Life Technologies GmbH, Darmstadt, Germany), respectively. Chromosome preparations were post-fixed with 4% formaldehyde after treatment with 0.05mg ml^–1^ pepsin in 0.01M HCl, washed in 2× SSC [0.03M Na-citrate (pH 7.0), 0.3M NaCl], and dehydrated through an ethanol series. The hybridization mixture containing probes, 50% de-ionized formamide, 10mM TRIS-HCl (pH 8.0), 1mM EDTA, and 2× SSC was dropped on the slides, overlaid by coverslips, and denatured for 2min at 80 ºC. After hybridization overnight at 37 ºC, the slides were washed in 2× SSC for 5min at room temperature and for 20min at 55 ºC, followed by dehydration through an ethanol series. Chromosomal DNA was stained by 1 µg ml^–1^ 4',6-diamidino-2-phenylindole dihydrochloride (DAPI) in Vectashield (Vector Laboratories, Inc., Burlingame, CA, USA).

### Barley transformation, the generation of the recipient line, and its re-transformation for gene targeting

The recipient construct p6Um was transformed into *Agrobacterium* strain AGL1 (Wang and Waterhouse, 2000), and barley (*H. vulgare* cv ‘Golden Promise’) was transformed via *Agrobacterium*-mediated gene transfer to immature embryos essentially as described previously (Hensel *et al.*, 2009). After co-culture, immature embryos were selected on 20mg l^–1^ hygromycin for 2 weeks in the dark followed by subculture on callus induction medium with 3mg l^–1^ bialaphos. Shoots were generated on medium with 4mg l^–1^ bialaphos and afterwards transferred on medium with 5mg l^–1^ bialaphos.

The donor constructs PAT-I-SceI/p6U-pro, PAT /p6U-pro, and pWBVec10 (Wang and Waterhouse, 2000) were transformed into *Agrobacterium* strain AGL1 to obtain strains A35, A37, and Vec10. For the gene targeting assays, homozygous BG212E29 plants were grown on soil in a controlled-environment chamber and spikes with immature caryopses were harvested. Immature embryos were dissected and co-cultivated with A35, A37, and Vec10 as described (Tingay *et al.*, 1997; Jacobsen *et al.*, 2006). Transformants were selected on 50mg l^–1^ hygromycin.

### Molecular analysis of gene targeting

Genomic DNA was prepared from young seedlings using the Qiagen Plant DNA easy kit (Qiagen, Hilden, Germany) as described by the manufacturer. PCR analysis for fragments <1.5kb was with regular PCR using Ampliqon *Taq* polymerase (Ampliqon A/S, Odense, Denmark) and long template PCR for the remainder with the Expand Long Template kit (Roche, Roche Diagnostics Deutschland GmbH, Mannheim, Germany) according to the manufacturer’s instructions. The primers were: Pubi_sense2 (TTCCGGTCCATGGTTAGG), P6u_sense (GATCGGCTCTAGTAGTCTGCAG), Hyg_anti-1 (CATTGTCC GTCAGGA CATTG), Hyg_anti-2 (TATTGACCGATTCC TTGCG), anti_SceI (TTATCCCTACTCCTC CATGG), and, for Supplementary Fig. S2 available at *JXB* online, BR-in3 (GGCGG GAAACGACAATCTGATC), pcrPATout-2-1 (TCGATGTAGTG GTTGACGATG), I-SceI-sense (CATGAATCTCGG ACCTAA CTC), and I-SceI-anti (CATTTCAGGAACGTCTCAGAG).

For the gel blot analysis, genomic DNA was digested with the restriction enzymes indicated in Fig. 4, the digest purified by phenol/chloroform extraction, and separated by agarose gel electrophoresis (0.8% agarose). Blotting and hybridization with a radioactively labelled *hpt* gene probe, a *Bgl*II/*Sac*I fragment isolated from pUC18/Hyg (Zobell, 2006), was as described (Markmann-Mulisch *et al.*, 2007).

## Results

### A transgenic target locus to analyse gene targeting in barley

Gene targeting is a method that uses sequence homology and HR to drive integration of a foreign sequence at a pre-determined position in the genome. Background information on gene targeting in plants is available in several reviews (Reiss, 2003; Zobell and Reiss, 2010; Puchta and Fauser, 2013) and the literature therein. In the variant almost exclusively used nowadays, an endogenous gene is replaced by an *in vitro* modified copy (gene replacement). Ideally, this process leads to seamless replacement of the resident (target or recipient) gene by the one introduced with the donor or repair construct. However, processes other than replacement can generate almost identical products, and a rigorous testing is necessary to ensure that integration had occurred at the target gene. With the exception of *Physcomitrella patens*, the efficiency of gene replacement in plants is extremely low. This necessitates assay systems in which a dominant, directly and easily selectable trait indicates replacement early in the experiment.

**Fig. 1.**
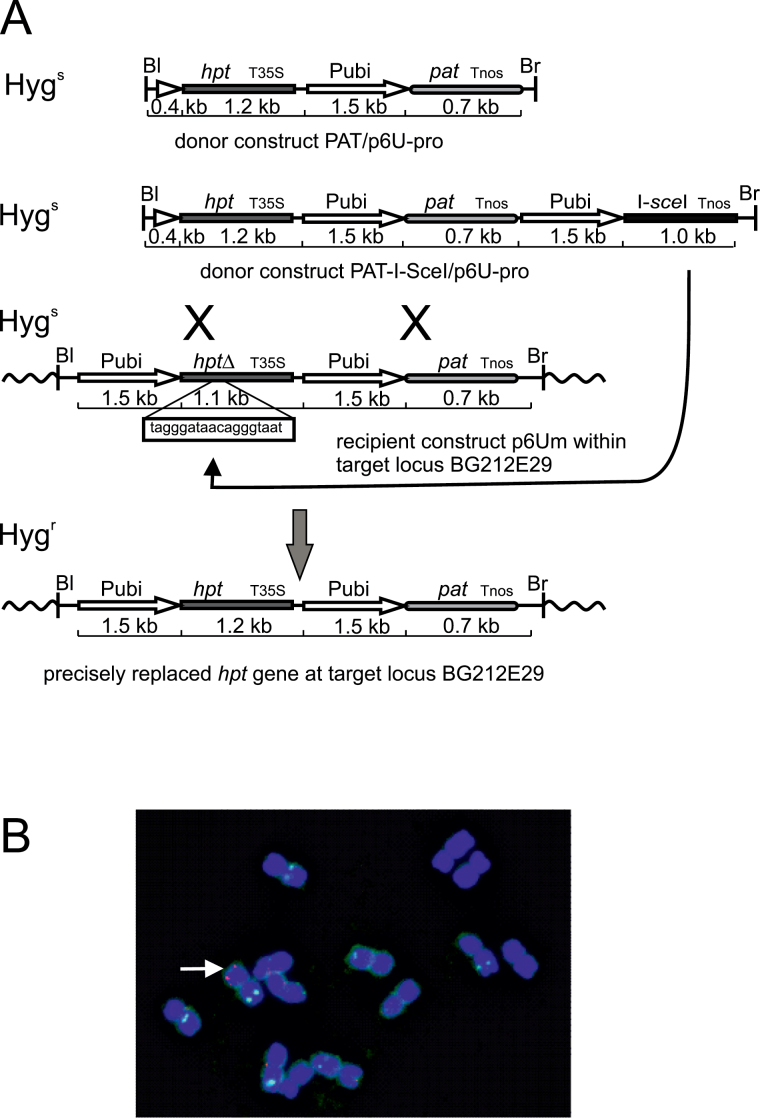
The gene targeting strategy and the transgenic target locus. (A) The scheme shows the recipient construct p6Um in the centre of the sketch after integration into the barley genome to form the target locus in BG212E29. The donor constructs PAT/p6U-pro and PAT-I-SceI/p6U-pro are shown above and the recombination product predicted for precise gene replacement below. In this design, transformation leads to transient I-*Sce*I expression. Cleavage of the I-*Sce*I site in the recipient induces recombination, thereby converting a defective into a functional *hpt* gene that confers resistance to hygromycin. The left (Bl) and right (Br) T-DNA borders are symbolized with a short vertical line, the intron which is part of the *ubiquitin* promoter by an open triangle, the *hygromycin phosphotransferase* (*hpt*) gene including the CaMV 35S terminator (T35S) by a black rectangle, the *ubiquitin* promoter (Pubi) by an open arrow, the *phosphinotricin acetyltransferase* (*pat*) gene including the *nopaline synthase* gene terminator (Tnos) by a round-edged, grey-shaded rectangle, and the I-*Sce*I coding region (I-*sce*I) including the *nopaline synthase* gene terminator (Tnos) by a black rectangle. The wavy lines symbolize neighbouring genomic sequences. The insertion of the I-*Sce*I site in the defective *hpt* gene (*hpt*Δ) of p6Um is shown, and the position symbolized by a white rectangle. The size of the individual segments is given. Hyg^s^ denotes sensitivity and Hyg^r^ resistance to hygromycin. (B) FISH analysis of plant BG212E29. The picture shows a complete metaphase with signals for 5S rDNA loci (green) and p6Um (red). Arrow points to the hemizygous insertion of p6Um.

The assay system (Fig. 1A) used here to study gene targeting with and without DSB induction is based on the conversion of a defective (*hpt*Δ) *hygromycin phosphotransferase* (*hpt*) resistance gene into an active hygromycin resistance-conferring version by replacing it with an intact, but promoter-less copy. The modification of *hpt*Δ consisted of a small deletion in the N-terminal part of *hpt* and the insertion of an I*-Sce*I recognition sequence at this position. This makes *hpt*Δ 68bp smaller than *hpt* in the donors, renders the *hpt* coding region non-functional, and provides the site for DSB induction in the target locus. To establish the plant carrying the target locus, the binary vector p6Um was constructed. This vector carries the recipient construct comprising the *hpt*Δ gene fused to the promoter and the first intron of the maize *Ubi-1* gene (*ubiquitin* promoter) (Christensen and Quail, 1996) followed by an *ubiquitin* promoter-driven *phosphinotricin acetyltransferase* (*pat*) gene. This gene confers resistance to the herbicides phosphinotricin (PPT), bialaphos, or Basta, and was used to select transformants. Transformed plants carrying a single locus of the recipient construct were selected by segregation, PCR, and DNA gel blot analyses, and the transgene was mapped by FISH. Finally, plant BG212E29 harbouring the target locus on the long arm of chromosome 2 (Fig. 1B) was chosen. Homozygous progeny were produced by self-pollination and the seeds were grown to obtain the material for subsequent re-transformation as detailed below.

### Analysis of gene replacement at the BG212E29 locus

Gene replacement was analysed with two different donor constructs. To achieve efficient gene replacement, both constructs were identical in sequence to the target gene over a region of 3.8kb which is only interrupted by the small insertion/deletion around the I-*Sce*I site in p6Um. The construct not allowing DSB induction was PAT/p6U-pro (Fig. 1A). This vector carries the intact but promoter-less *hpt* gene followed by the *pat* gene under *ubiquitin* promoter control. The donor construct allowing DSB induction is PAT-I-SceI/p6U-pro (Fig. 1A). This vector is identical to PAT/p6U-pro in the region of homology to the recipient, but carries an additional segment at the end that encodes a codon-optimized *ubiquitin* promoter-driven I-*Sce*I gene (Vu *et al.*, 2014). Transformation with this construct allows transient expression of the I-*Sce*I gene and consequently induction of a DSB at the I-*SceI* site in the recipient.

In total, four independent transformation experiments were performed, two each with PAT-I-SceI/p6U-pro (strain A35 in transformations UB53 and UB54) and PAT/p6U-pro (strain A37 in transformations UB56 and UB57). Transformants were selected on hygromycin. Each experiment included pWBVec10 carrying an expressed, intact *hpt* gene as transformation control and reference. A transformed immature embryo was counted as a transformant when at least one shoot that rooted on hygromycin was obtained from it. However, usually more than one shoot was obtained from one callus. Because shoots derived from one callus may represent independent transformants, all shoots were included in the analysis.

The results of these experiments (Table 1) showed that the efficiency of barley transformation was heavily dependent on the plant material and varied for the transformation standard pWBVec10 between 20% and 70%. To obtain the efficiencies in comparison with that achievable with an intact *hpt* gene, re-transformation efficiencies relative to pWBVec10 were calculated (Table 1). The donor construct PAT-I-SceI/p6U-pro yielded 56% and PAT/p6U-pro 19% of the transformants that the pWBVec10 reference (set to 100%) would have produced. This number of hygromycin-resistant individuals was higher than expected for gene targeting. In both experiments, the donor allowing DSB induction produced more transformants, suggesting that DSB induction contributes to the establishment of a functional *hpt* gene.

**Table 1.** Transformation experiments to analyse gene targeting at the BG212E29 locus

	PAT/p6U-pro				PAT:I-SceI/p6U-pro			
	Experiment UB56		Experiment UB57		Experiment UB53		Experiment UB54	
*Agrobacterium* strain	Vec10	A37	Vec10	A37	Vec10	A35	Vec10	A35
Embryos transformed	20	110	20	170	20	180	20	140
No. of independent transformants obtained	14	15	12	18	10	50	4	16
No. of shoots obtained	35	20	29	28	25	66	8	29
Transformation efficiency	70%	14%	60%	11%	50%	28%	20%	11%
Transformation efficiency relative to Vec10		19%		18%		56%		57%

### PCR analysis of primary transformants

**Fig. 2.**
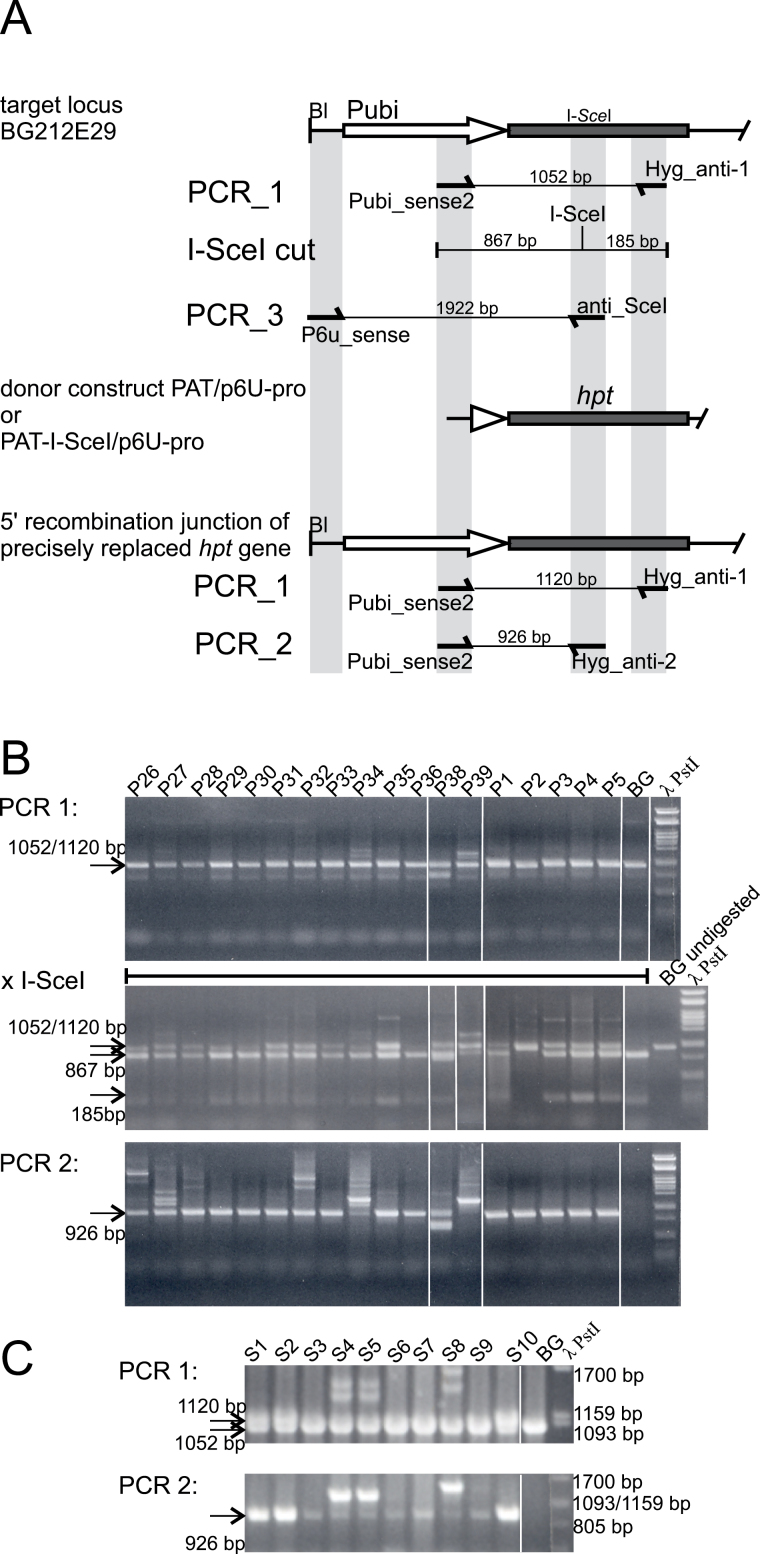
PCR analysis of transformants. (A) The PCR strategy to analyse gene replacement is shown. The region in the BG212E29 target locus involved in the formation of the 5' recombination junction is shown on the top. Below are the corresponding regions of the donor and of a precisely formed junction. PCR primers are shown by arrows pointing in the direction of synthesis. The size of the PCR products is given. (B) Representative results obtained by PCR screening of primary re-transformants obtained with PAT-I-SceI/p6U-pro in UB53. DNA of the plants as indicated on top of lanes was amplified by PCR_1 and PCR_2. In addition, PCR_1 products were digested by I-*Sce*I. DNA was separated by agarose (1.5%) gel electrophoresis, and the gels were stained with ethidium bromide and photographed. The DNA size standard in the rightmost lane is *Pst*I-digested phage λ DNA. BG, BG212E29 target locus line. (C) Results obtained by PCR screening of primary re-transformants SR1–SR10 obtained with PAT/p6U-pro in experiment UB57. DNA was amplified by PCR_1 and PCR_2, as above. Products obtained in PCR_1 were separated by high-resolution and those of PCR_2 by standard (1.5%) agarose gel electrophoresis. The DNA size standard in the rightmost lane is *Pst*I-digested phage λ DNA. The fragment sizes in the visible part of the DNA standard are given at the right end of the gel picture. BG, BG212E29 target locus line.

Gene replacement in our system forms an intact *hpt* gene under *ubiquitin* promoter control at the target locus. This recombination product is found by a PCR approach designed to detect either the final product or specifically the 5' recombination junction formed between the donor and target locus upon HR. The first one (PCR_1) detects the modified and/or the unmodified target gene using a primer combination (Fig. 2A) starting in the *ubiquitin* promoter outside of the region homologous to the donor construct (primer Pubi_sense2) and ending within the *hpt* encoding region downstream of the I-*Sce*I insertion site (Hyg_anti-1). The modified and unmodified fragments differ in size by 68bp and by the presence of the I-*Sce*I site in the unmodified version. In the second approach (PCR_2), one primer was exclusively present in the target (Pubi_sense2) and the other in the donor (Hyg_anti-2) (Fig. 2A). PCR_2 detects only the modified target gene and also larger recombination junctions that would be difficult to see with PCR_1.

The re-transformants were screened for potential gene replacement events using both approaches. Re-transformants for PAT-I-SceI/p6U-pro were obtained from experiment UB53. The corresponding data are listed in Table 2A and representative examples are shown in Fig. 2B. The diploid homozygous BG212E29 target line carries two target locus alleles, one or both of which can be modified by gene targeting. The expected fragment of ~1kb in PCR_1 obtained from unmodified as well as from modified target loci was digested with the restriction enzyme I-*Sce*I which cleaves the fragment derived from the unmodified locus asymmetrically in two. The approximately equimolar ratio of resistant and sensitive fragments expected for a mono-allelic gene replacement was present in about one-third of the re-transformants; in six cases the fragment was entirely resistant to cleavage, and sensitive or mostly sensitive in the remaining plants. Plants with a potentially mono-allelic replacement were considered as prime candidates. PCR_2 yielded a fragment with all analysed plants, although the yield was quite low in some cases. The size of the fragment was that for a correct junction (926bp) in most plants, but was larger in a number of other plants (oversize junction). Sequencing of representative PCR_1 and PCR_2 products confirmed that the amplified products corresponded to the predicted sequences. The DNA sequence of an oversize fragment showed that a donor T-DNA had integrated into the intact *ubiquitin* promoter of another T-DNA such that the *hpt* gene sequence was brought under *ubiquitin* promoter control.

**Table 2.** Summary of PCR analyses (A) Re-transformants obtained with PAT-I-SceI/p6U-pro

1	Primary plant no.	**P1**	**P2**	**P3**	**P4**	**P5**	P6	P7	P8	P9	P11	P12	P13	P14	P15
2	from callus no./shoot no.	11/1	6/1	7/1	9/2	9/3	10/1	10/3	14/2	2/1	3/2	4/1	4/2	12/1	12/2
3	Fragments in PCR_1 (kb)	~1	~1	~1	~1	~1	~1	~1	~1	~1	~1	~1	~1	~1	~1
4	PCR_1 I-*Sce*I digested (%)	75	0	50	75	75	100	100	100	100	100	100	100	50	100
5	Fragments in PCR_2 (kb)	0.9	0.9	0.9	0.9	0.9	0.9	0.9	~1.5	~1.5	~1.5	None	0.9	0.9	0.9
6	In segregation analysis as		R1											R6	
1		P16	P17	P18	P19	P20	P21	P22	P23	P24	P25	**P26**	**P27**	**P28**	**P29**
2		15/1	15/2	16/3	17/1	18/1	18/2	19/1	20/1	20/2	21/1	22/1	23/2	24/1	25/1
3		~1	~1	~1	~1	~1	~1	~1	~1	~1	~1	~1	~1	~1	~1
4		100	100	100	50	100	100	75	100	50	100	75	75	75	100
5		~1.0/1.7	~1.0/1.7	0.9	0.9	0.9/~3.0	0.9	0.9	0.9	0.9	~1.3	0.9/~5.0	0.9/~1.5	0.9	0.9
6														R7	
1		**P30**	**P31**	**P32**	**P33**	**P34**	**P35**	**P36**	**P38**	**P39**	P40	P41	P42	P43	P44
2		25/2	26/1	26/3	27/1	28/1	28/2	29/1	31/1	31/2	32/1	34/1	34/2	37/1	38/1
3		~1	~1	~1	~1	~1	~1	~1	~1 /~0.8	~1/1.3	~1	~1	~1	~1	~1
4		100	50	75	100	100	50	100	50	0	0	0	50	100	0
5		0.9	0.9	0.9/~4.0	0.9	~1.3	0.9	0.9	0.9/~0.8	~1.3	0.9	0.9	0.9/~3.0	0.9/~3.0	0.9
6			R8						R2	R3					
1		P45	P46	P47	P48	P49	P50	P51	P52	P53	P54	P55	P56	P57	P58
2		38/2	38/3	40/1	42/2	43/1	45/1	45/2	47/2	48/5	48/2	49/2	50/1	51/1	51/2
3		~1	~1	~1	~1	~1	~1	~1	~1	~1	~1	~1	~1	~1	~1
4		50	75	100	100	50	75	50	50	50	50	50	0	50	100
5		0.9	0.9	0.9	0.9/~3.0	0.9	0.9	0.9	0.9	0.9	0.9	0.9	~1.7	0.9	0.9
6		R4							R9	R10			R5		

Line 1: selection of primary re-transformants obtained in transformation experiment UB53 and analysed by PCR. Line 2: list of the corresponding callus and shoot numbers from which the individual re-transformants were derived. Line 3: the approximate size of the product(s) obtained in PCR_1. Line 4: results obtained by I-*Sce*I digestion of PCR_1 products. The digests were classified as 0% when the fragment was resistant or largely resistant to digestion, 50% when undigested and digested product were present in approximately equal amounts, 75% when more digested than undigested product was obtained, and 100% when only digested product was present. Line 5: the size of product(s) obtained in PCR_2. Line 6: designation of plants finally selected for segregation analysis.

Primary re-transformants printed in bold are shown in Fig. 2.

PCR products were analysed by standard agarose gel electrophoresis.

**Table:** (B) Re-transformants obtained with PAT/p6U-pro

1	Primary plant no.	**S1**	**S2**	**S3**	**S4**	**S5**	**S6**	**S7**	**S8**
2	from callus no/shoot no	UB56 1/1	UB56 4/3	UB56 7/1	UB56 7/3	UB56 7/5	UB56 7/6	UB56 10/2	UB56 10/3
3	Fragment 1 in PCR_1 (kb)	1.052	1.052	1.052	1.052	1.052	1.052	1.052	1.052
4	Fragment 2 in PCR_1 (kb)	1.120	1.120	-	~1.3/1.5	~1.3/1.5	-	-	~1.4/1.6
5	Fragments in PCR_2 (kb)	0.926	0.926	(0.926)	~1.2	~1.2	(0.926)	(0.926)	~1.5
6	In segregation analysis as								
1		**S9**	**S10**	S11	S12	S13	S14	S15	
2		UB56 13/1	UB56 14/1	UB57 7/1	UB57 11/1	UB57 3/1	UB57 2/1	UB57 3/2	
3		1.052	1.052	1.052	1.052	1.052	1.052	1.052	
4		-	1.120	1.120-	-	1.120	1.120	1.120	
5		(0.926)	0.926	0.926	-	0.926	0.926	0.926	
6				SR1		SR2		SR3	

Line 1: selection of primary re-transformants obtained in transformation experiments UB56 and UB57 and analysed by PCR. Line 2: list of the corresponding callus and shoot numbers from which the individual re-transformants were derived. Line 3: the size of the smallest fragment in PCR_1. A 1.052kb fragment indicates the presence of the unmodified target locus. Line 4: the size of the larger fragments in PCR_1. A 1.120kb fragment indicates the presence of a 5' recombination junction; larger fragments the presence of T-DNA in T-DNA integrations. Line 5: size of product(s) obtained in PCR_2. A 0.926kb fragment indicates the presence of a 5' recombination junction; larger fragments the presence of T-DNA in T-DNA integrations. Line 6: designation of plants finally selected for segregation analysis.

Primary re-transformants printed in bold are shown in Fig. 2.

PCR_1 products were analysed by high-resolution, PCR_2 products by standard agarose gel electrophoreses.

The size of defined fragments is given as an exact number, others as an estimate (~).

A number in parentheses indicates a weak signal.

The PCR results for 15 plants (S1–S15) obtained with PAT/p6U-pro are shown in Table 2B and representative examples in Fig. 2C. PCR_1 fragments were separated by high-resolution gel electrophoresis that allows discrimination of the products derived from the unmodified (1.052kb) and modified (1.120kb) target loci. This analysis suggested that transformation with both donors resulted in largely the same type of transformants. A large number of re-transformants had oversize junctions or possessed the unmodified locus exclusively. Nevertheless, a substantial number represent potential, mono-allelic gene replacements. However, it is in principle impossible to prove gene replacement in primary re-transformants since ectopic targeting (Hanin *et al.*, 2001; Reiss, 2003) and extrachromosomal HR (Offringa *et al.*, 1990; Degroot *et al.*, 1994) cannot be excluded (see below). Such cases display a 5' recombination junction or an intact *hpt* gene that is indistinguishable from gene replacement by PCR in primary transformants. Therefore, additional data are needed to confirm gene replacement.

### Targeted modifications are stably inherited as a single Mendelian gene

**Fig. 3.**
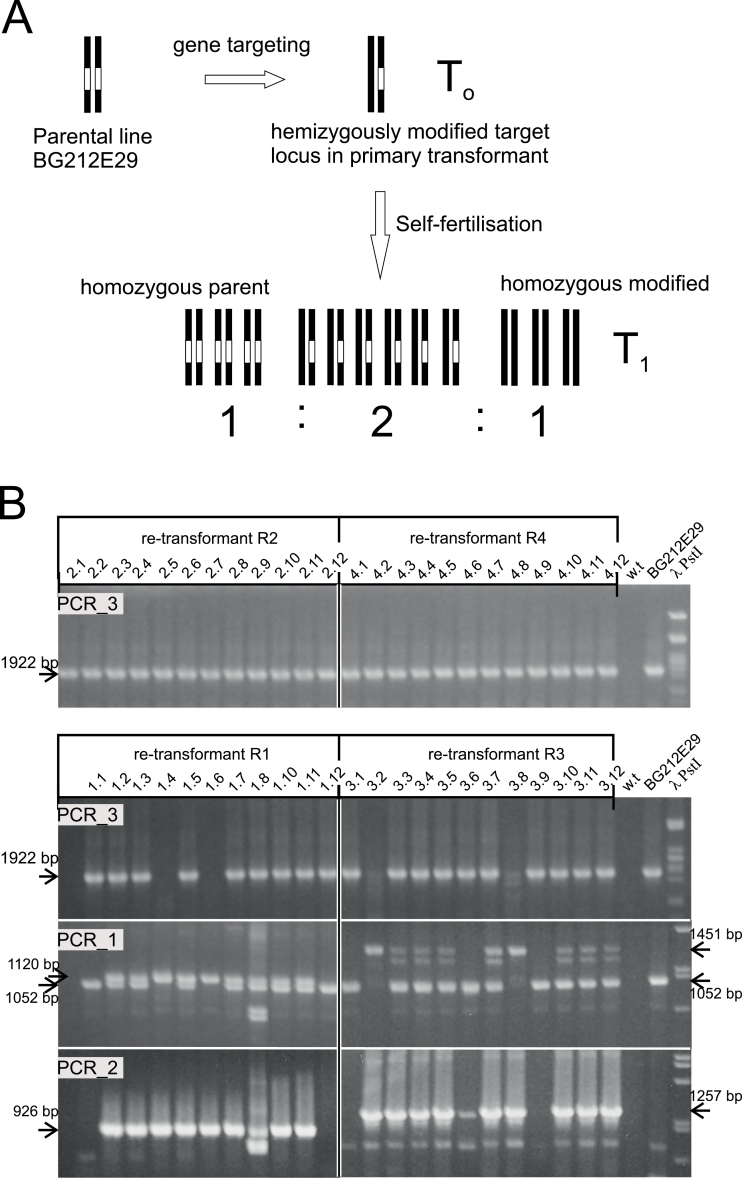
PCR analysis of selected candidate PAT-I-SceI/p6U-pro re-transformants. (A) The rationale of the analysis. The homozygous parental BG212E29 line carries two target locus alleles, symbolized by the two chromosomes carrying the I-*Sce*I recognition sequence (*hpt*Δ) shown as the white rectangle within the *hpt* gene in the target locus. Gene replacement modifying one allele promotes conversion of *hpt*Δ into *hpt*. These two alleles segregate as a single Mendelian trait in a 1:2:1 ratio of homozygous target locus, and hemizygous and homozygous modified locus upon self-fertilization. (B) Results of the analysis of a segregating population of siblings obtained with four of the 10 R1–R10 candidate plants. PCR_3 detects the presence of an unmodified target locus. DNA of 12 siblings of each progeny was amplified by PCR_3, the products were separated by agarose gel electrophoresis, and the gels were stained with ethidium bromide and photographed. The analysis shows the absence of an unmodified target locus in siblings of plant R1 and R3. DNA of the same plants was amplified by PCR_1 and PCR_2, and the products separated by high-resolution (1.5%) and standard agarose gel electrophoresis, respectively. PCR_1 and PCR_2 in these plants confirm the absence and presence of unmodified and modified loci as expected for Mendelian segregation.

A hallmark of gene replacement is modification of the target locus at its position in the genome, in contrast to ectopic targeting or extrachromosomal recombination. For mono-allelic gene replacement, self-fertilization of the primary transformant yields homozygous individuals in the progeny. The exclusive presence of the modified locus in this segregating population will definitively prove gene replacement (Fig. 3A).

Ten PAT-I-SceI/p6U-pro individuals (R1–R10, Table 2A) obtained with DSB induction were selected for segregation analysis. Six of them (R2, R4, R6, R8, R9, and R10) were prime candidates with a potential mono-allelic gene replacement. In addition, PCR_1 had indicated that six candidates existed with a potential bi-allelic modification. Three of them (R1, R3, and R5), of which R3 and R5 showed an oversize PCR_2 junction fragment, were also included. Due to limited plants with sufficient seed set, one unlikely candidate (R7) was also chosen. The replacement of *hpt*Δ by the promoter-less *hpt* eliminates the deletion/insertion carrying the I-*Sce*I recognition site in the BG212E29 target locus. This feature was used to detect individuals in which the unmodified target locus was absent in the segregating population. The corresponding PCR used a sense primer (p6u_sense) located in the left T-DNA border region of the BG212E29 target line in combination with the antisense primer anti-SceI that binds within the insertion carrying the I-*Sce*I recognition sequence in *hpt*Δ (PCR_3, Fig. 2A).

A population of 12 seedlings, or as many as available, obtained from each of these plants was screened by PCR_3. The 1.9kb fragment indicative of the presence of the unmodified BG212E29 target locus was detected in the progeny of all primary re-transformants except some of R1 and R3 (Fig. 3B). This fragment was missing in two out of 11 individuals of R1 and two out of 12 individuals of R3. In a non-chimeric transformant, a locus modified in one allele will segregate in a self-pollinated progeny as a Mendelian trait and produce a quarter of progeny with a homozygously modified locus (Fig. 3A). To confirm further Mendelian segregation, the segregating progeny of these two plants were analysed additionally by PCR_1 and PCR_2 (Fig. 3B). Analysis of PCR_1 products on high-resolution agarose gels showed that the two PCR_3-negative siblings of R1, plants R1.4 and R1.6, exclusively had a modified target locus. This result was confirmed by PCR_2 which showed in addition that the 5' recombination junction was correct. Among the PCR_3-positive siblings were two which exclusively displayed the target locus fragment (plants R1.1 and R1.12) in both PCR_1 and PCR_2, as expected for individuals with a homozygously unmodified target locus in the population. The remaining siblings were hemizygous, also shown by PCR_1 and PCR_2. This is the result predicted for the replacement of a single allele in the homozygous BG212E29 target locus line and its Mendelian inheritance.

The analysis for plant R3 showed basically the same result, except that the 5' recombination junction-specific fragment obtained with PCR_2 was larger than predicted for precise HR. The PCR_1 and PCR_2 results for the two PCR_3-negative plants (R3.2 and R3.8) showed the modified locus and the absence of the unmodified target locus. Therefore, these plants are homozygous for the targeted locus. Three PCR_3-positive siblings (R3.1, R3.6, and 3.9) exclusively displayed the unmodified target locus, shown by PCR_1 and PCR_2, and are homozygous for the unmodified target locus. The remaining siblings had both a modified and an unmodified locus and thus are hemizygous. To determine the structure of the 5' recombination junction of plant R3, the PCR_2 product of sibling R3.2 was sequenced. The DNA sequence showed that this junction was generated by insertion of a T-DNA into the *ubiquitin* promoter region upstream of the *hpt* gene of the target locus sequence (Supplementary Fig. S1B at *JXB* online). The insertion site is 188bp downstream of the Pubi_sense2 primer-binding site and contained the complete T-DNA left border sequence, as predicted for a canonical T-DNA integration. This position is within the intron which is part of the *ubiquitin* promoter, and this sequence is likely to be spliced out to allow efficient expression of the replacement *hpt* gene of the donor construct. The DNA sequence of the other supposedly unmodified target locus allele of plant R3, obtained by PCR_1 from sibling R3.1, showed that actually this one was also modified (Supplementary Fig. S1A). The sequence revealed an 8bp deletion in the I-*Sce*I recognition sequence which was not detectable by PCR. This deletion is next to the cleavage site and therefore was probably generated in the primary re-transformed plant by I-*Sce*I-mediated DSB induction and subsequent NHEJ repair.

**Fig. 4.**
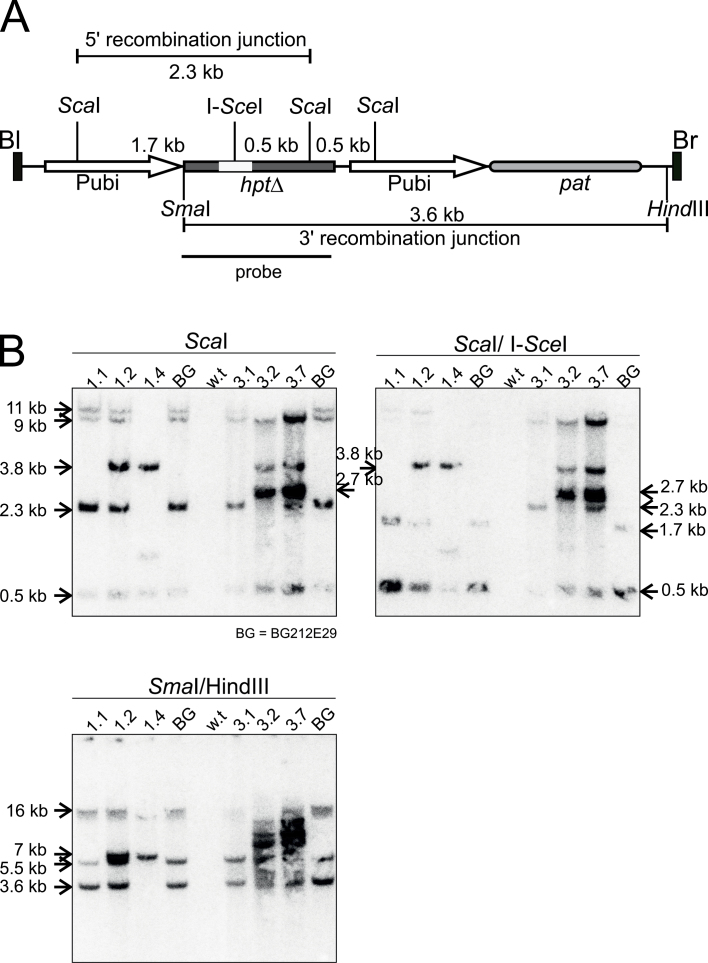
Confirmation of gene replacement by DNA gel blot analysis. (A) The scheme shows the BG212E29 target locus together with the recognition sites of the restriction enzymes used. The fragment sizes are shown below together with the *hpt* gene probe used for hybridization. Symbols are as in Fig. 1. (B) DNA of the plants indicated was digested with the restriction enzymes shown on top of the panels. DNA was separated by agarose gel electrophoresis, blotted to a membrane, and the membrane was hybridized to the radioactively labelled *hpt* gene probe. The size of relevant fragments is shown to the left and right of panels. Sizes are derived from a *Pst*I-digested phage λ DNA size standard co-migrating on the original agarose gel and are given as an exact number when they corresponded to predicted values, otherwise as a size estimate. BG, BG212E29, w.t., wild-type Golden Promise DNA.

The PCR results obtained for plants R1 and R3 were confirmed by DNA gel blot analysis (Fig. 4). The 5' recombination junction is located on a 2.3kb *Sca*I fragment (Fig. 4A). This fragment also covers the I-*Sce*I site in the target locus. Upon replacement of *hpt*Δ by the promoter-less *hpt*, the I-*Sce*I site is lost and the fragment becomes 68bp longer, but this difference is not resolved by the gel analysis. Genomic DNA of one homozygously unmodified, one homozygously modified, and one hemizygously modified individual, as determined by the PCR, was digested with *Sca*I and *Sca*I/I*-Sce*I, and the blotted membrane was hybridized using a probe detecting the entire *hpt* gene (Fig. 4B). The *Sca*I digest confirmed the presence of a correct *hpt*Δ gene copy in BG212E29, and the corresponding 2.3kb fragment was sensitive to I-*Sce*I digestion. The same fragment was present in the homozygous unmodified and the hemizygous progeny of plants R1 and R3. This fragment was I-*Sce*I sensitive in R1 individuals and resistant in those of plant R3, in accordance with the PCR and DNA sequence data. Most importantly, the fragment diagnostic for the unmodified target locus (*Sca*I and/or *Sca*I/I*-Sce*I) is absent in the homozygously modified individuals R1.4 and R3.2, confirming targeted modification in plants R1 and R3. However, the fragment indicating a precise 5' recombination junction (2.3kb I*-Sce*I-resistant *Sca*I fragment) is absent in both plants. This is expected for plant R3 as the PCR and the DNA sequence predicted a larger 5' recombination junction and the 2.7kb fragment detected in the gel blot analysis is in accordance with these data. In plant R1, the size of this fragment is ~3.8kb instead of the expected 2.3kb. Since PCR_1 and PCR_2 showed a precise 5' recombination junction for this plant, this result suggests additional rearrangements or insertions within the replaced segment. There are two more fragments in the target locus that should be detected by the probe, a 0.5kb *Sca*I fragment and the second *Sca*I/I*-Sce*I fragment of 0.5kb, both located towards the 3' end of the *hpt* gene. Fragments of 0.5kb are present in all digests from all plants, and are predicted from the structure of the recipient construct p6Um and the insertion of a complete T-DNA copy at the target locus. The presence of an intact target locus copy in BG212E29 was also confirmed by PCR (Supplementary Fig. S2A at *JXB* online, and below) using primers spanning the entire T-DNA region. However, additional and unexpected fragments are detected using the probe. The corresponding bands could be the results of partial digests, caused, for example, by the existence of a non-digestible DNA fraction in the genome due to methylation of restriction sites. However, since these fragments occur in the digests of all restriction enzymes, probably additional partial or rearranged p6Um T-DNA sequences are inserted at or close to the target locus.

The *hpt* gene together with the adjacent *pat* gene is located on a 3.6kb *Sma*I/*Hin*dIII fragment that covers the 3' recombination region and ends close to the T-DNA right border (Fig. 4A). The 68bp size difference between *hpt*Δ in the target locus and the modified *hpt* gene is not resolved in the analysis. Therefore, this fragment should not change upon precise replacement. When BG212E29 DNA was digested with *Sma*I and *Hin*dIII, two fragments of 3.6kb and ~5.5kb were obtained. The 3.6kb band is the expected target locus fragment. PCR (Supplementary Fig. S2A at *JXB* online, and below) has shown that a complete and intact p6Um insert is present at the target locus. Therefore the ~5.5kb band represents the *Hin*dIII fragment in the digest that was resistant to *Sma*I, most probaby due to partial CpG methylation at the recognition site. These target locus fragments are absent in the homozygous targeted individual (R1.4) of plant 1 and replaced by an ~7kb fragment. This confirms that the insertion is aberrant regarding the replaced sequence towards the 3' recombination junction. In contrast, 3.6kb and 5.5kb fragments are present in the homozygous targeted individual of plant R3 (R3.2), showing that the 3' recombination border is precise in this plant. However, quite a number of additional fragments are present, as already seen in the *Sca*I and the *Sca*I/I*-Sce*I digests. Therefore, plant R3 contains (an) additional donor construct T-DNA insertion(s) co-segregating with the targeted insertion. The presence of additional donor construct insertions in plant R3 suggests that a fully functional I-*Sce*I gene is also present in this plant. To confirm this assumption and to analyse additional plants, five primary retransformed plants were analysed by PCR. This analysis by an I-*SceI* coding region-specific PCR (primer pair SceI-sense/SceI-anti) showed that at least plants R1–R4 had an intact I-*SceI* gene integrated in the genome (Supplementary Fig. 2B).

To analyse the rearrangements in plant R1 in more detail (Supplementary Fig. S2A at *JXB* online), the entire target locus sequence was amplified with a pair of primers located towards the left (p6u_sense) and close to the right (BR-in3) T-DNA border sequences. The 5.2kb product obtained with the BG212E29 target locus plant was of the predicted size, indicating that a complete p6Um T-DNA insert is present in the target locus. The same product was obtained with an individual of the plant R1 progeny homozygous or hemizygous for an unmodified target locus, but not with that with the homozygous modified locus. This result suggests that replacement has induced major deletions at the target locus. To analyse this in more detail, a PCR with the same p6u_sense primer and the antisense primer (PCR_PATout2-2-1) located within the *pat* gene was performed. The expected product of 4.4kb was obtained with BG212E29 and the offspring with an unmodified target locus, but not with the plant homozygous for the modified locus. These results suggested that the deletion had included the *pat* gene and the right border sequences of the target locus.

The eight non-targeted re-transformants obtained with PAT-I-SceI/p6U-pro were also analysed further by PCR_1 and PCR_2. In the population of siblings from prime candidates (R2, R4, R6, R8, R9, and R10), the PCR fragments originally found in the primary re-transformants were absent in three (R2, R6, and R8) and present in the other three. Two non-prime candidates also inherited the originally present fragments. These insertions segregated independently from the target locus, and no sibling was found that was homozygous for the fragment derived from the modified locus.

The siblings of the three prime candidates obtained without DSB induction (PAT/p6U-pro re-transformants SR1–SR3, Table 2B) that also potentially carried a mono-allelic gene replacement were analysed with PCR_1 and PCR_2. PCR_1 products of 12 siblings obtained from SR1 and SR2 and eight of SR3 were separated by high-resolution agarose electrophoresis (Supplementary Fig. S3 at *JXB* online). The fragment derived from the unmodified target locus was present in all siblings from all re-transformants. The other fragment segregated independently from it. These individuals were positive for the 5' junction-specific PCR_2. This result demonstrated that none of these re-transformants was targeted.

In summary, the data obtained with both donor constructs showed that many re-transformants were generated by the insertion of a donor into actively transcribed regions and that this caused activation of the *hpt* gene of the donor construct. The PCR_1 fragment of the unmodified target locus was solely present in these individuals and the 5' recombination junction fragment of the correct size in PCR_2 was probably generated in the PCR by template switch recombination (Judo *et al.*, 1998; De Semir and Aran, 2003), a common PCR artefact that is difficult to discriminate from true results. A number of other re-transformants carried T-DNA into T-DNA integrations. These events can also generate an expressed *hpt* gene from donor sequences already prior to integration into the genome. These individuals also had exclusively the PCR_1 fragment from the unmodified locus, but an oversize product in PCR_2. In the individuals with a pattern suggesting a mono-allelic gene replacement, originally selected as the prime candidates, the fragment from the modified locus segregated either independently of the target locus in the progeny or was not inherited. The inherited insertions can be generated after extrachromosomal HR (Offringa *et al.*, 1990; Degroot *et al.*, 1994) or by ectopic targeting (Hanin *et al.*, 2001; Reiss, 2003). Extrachromosomal HR could occur between homologous regions in the *ubiquitin* promoters present on both donor constructs and the intron in front of the promoter-less *hpt* gene (Fig. 1A) before random integration. In contrast, ectopic targeting is an alternative outcome of HR at the target locus. Following initiation of HR, target locus sequences are copied into the donor to form a 5' recombination junction and a product that is identical in structure at this end to the one created by replacement, but inserted at a random position. Ectopic targeting and extrachromosomal HR are indistinguishable by standard PCR approaches and segregation analysis. However, ectopic targeting is rare, and thus most of these events probably go back to extrachromosomal HR, with exceptions discussed below. In the three PAT-I-SceI/p6U-pro prime candidates which did not inherit the modifications, gene replacement induced by a DSB or possibly extrachromosomal HR could have occurred, but these events happened in a subpopulation of cells that finally did not enter the germline.

## Discussion

We analysed gene targeting in barley using a transgenic locus that allowed assessment of efficiencies in the presence and absence of DSB induction. This system allowed the use of an optimal tool for DSB induction, the meganuclease I-*Sce*I, and at the same time was designed for optimal homology between target and donor. In turn, we had to deal with background transformation inherent in such systems (Offringa *et al.*, 1990; Degroot *et al.*, 1994). The assay is based on the conversion of *hpt*Δ carrying the I-*Sce*I recognition site into an active *hpt* gene by gene replacement with a promoter-less *hpt* gene present in the donor constructs. Transformation of the target locus plant with both donors yielded an unexpectedly high number of re-transformants, whether DSBs were induced (PAT-I-SceI/p6U-pro re-transformants) or not (PAT/p6U-pro re-transformants). Analysis of both groups of re-transformants showed that the majority were generated by T-DNA into T-DNA integration or insertion into actively transcribed regions. A screen for gene replacement among potential prime candidates showed that these were often generated by extrachromosomal HR and subsequent random integration. However, re-transformants obtained with DSB induction contained six exceptional individuals with characteristics of bi-allelic target locus modifications. The segregation analysis of three of them (R1, R3, and R5) revealed that two of them were targeted (R1 and R3). The strategy to screen for gene replacement in PAT-I-SceI/p6U-pro re-transformants was based on the detection of the I-*Sce*I site in the target locus that discriminates it from the replaced *hpt* gene. However, this site can also be lost by targeted mutagenesis when this DSB is repaired by NHEJ instead of HR. Indeed, one of the targeted plants (R3) was a bi-allelic modification, with one allele carrying a targeted mutation and the other one the replacement. Bi-allelic modifications were probably also present in the parents of the other two, but R1 transmitted only one allele with gene replacement, and R5 none of the modified alleles, as discussed below. The modification of both alleles together in the same cell is a relatively rare event and likely to be the result of high activity of DSB induction. This suggests that the original screen has identified the few individuals among the re-transformants in which the I-*Sce*I expression levels were high and caused efficient DSB induction. These were the ones with targeted modifications. Many primary re-transformants probably carry an I-*Sce*I gene stably integrated in the genome, as suggested by an analysis of five of them (Supplementary Fig. S2B at *JXB* online). As a consequence, I-*Sce*I is continuously expressed, not only transiently during transformation. Continued expression implies that targeted mutations (and gene replacements, as long as free donors were available) had not necessarily occurred exclusively in the cell originally transformed, but may have been generated later in the developing callus and plant tissues derived from this cell. This process could generate chimeric plants in which different parts and tissues have different genomic modifications. Inheritance of a modified target sequence then depends on the presence in the subpopulation of cells that contribute to the germline. Limited inheritance of modifications present in primary re-transformants probably goes back to mosaicism, with subpopulations of cells differing in their genomic modifications. Failure to inherit modifications present in primary re-transformants was tentatively also observed with other plants (R2, R6, and R8) obtained after DSB induction.

Targeted mutagenesis does not occur in the absence of DSB induction, and bi-allelic gene replacement is rare. No gene targeting event was detected in the selection of prime candidates with a potential mono-allelic gene replacement, neither from PAT-I-SceI/p6U-pro nor from PAT/p6U-pro transformants. This observation suggests that gene replacement increases with DSB induction efficiency. Such individuals do not exist among the PAT/p6U-pro re-transformants. Therefore, gene replacement probably did not occur in the re-transformants obtained without DSB induction. Moreover, we found a frequency of targeting in barley well below 1 in 1000 transformants (U. Breier e*t al*., unpublished results) using an assay system like that of Endo *et al.* (2007) in rice, operating without DSB induction. In the present experiments (Table 1), we observed three times more transformants with than without DSB induction. This increase in transformants suggests a significant contribution of DSB induction, although it is not fully reflected in the number of gene replacements detected. The reasons why could be: (i) there were more replacement events in the population of primary transformants, but these escaped detection in the screen; (ii) targeting occurred more frequently, but many events were not transmitted to the germline and therefore were not present in the next generation; (iii) a number of events with a correct 5' recombination junction were actually ectopic targeting and not extrachromosomal HR; or (iv) DSB induction could have stimulated HR and consequently also extrachromosomal HR.

**Fig. 5.**
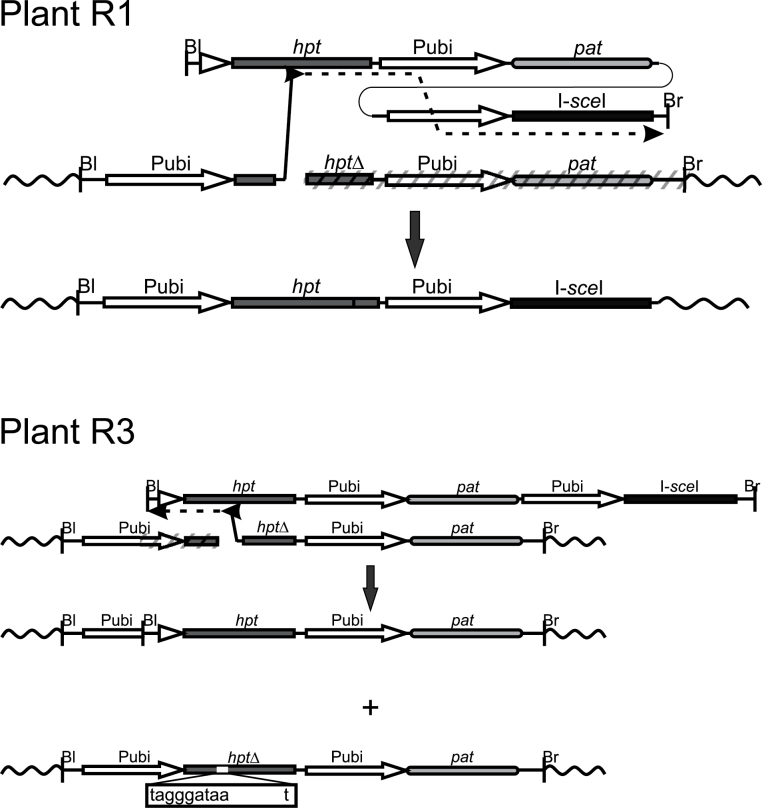
Gene replacement models for plants R1 and R3. The details are given in the text, and symbols are as in Fig. 1. In addition, a solid line with a filled arrowhead indicates a single-stranded DNA end initiating strand invasion. A broken line indicates replicated DNA, and cross-hatched areas indicate DNA degradation by end resection.

The two targeted plants were both produced by one-sided integration [i.e. the donor fragment had inserted at one end by precise HR and at the other by NHEJ (Puchta *et al.*, 1996; Puchta, 1998; Reiss *et al.*, 2000)]. Plant R3 was selected originally as a plant with an aberrant 5' recombination junction. The left end of a donor construct T-DNA had inserted in this plant into the *ubiquitin* promoter in front of the *hpt*Δ target gene (Supplementary Fig. S1 at *JXB* online) while precise HR formed the 3' recombination junction. Synthesis-dependent strand annealing (SDSA) (Puchta, 1998) after invasion of the resected right end of the I-*Sce*I-generated DSB at the target locus DNA into donor plasmid sequences may have formed the 3' recombination junction (Fig. 5). Following re-synthesis of the donor up to the left T-DNA border, this end was inserted by NHEJ at the left end of the break which was resected up to the insertion point in the *ubiquitin* promoter upstream of the *hpt*Δ gene. Alternatively, DSB induction may have initiated integration of a donor construct T-DNA at the open break (Chilton and Que, 2003). Crossover HR within the long region of homology, between the break and the right border sequence in the target locus, could have promoted homology-directed integration of the donor at this end, while a canonical T-DNA integration into the upstream *ubiquitin* promoter sequences formed the other.

The integration for plant R1 seems to be more complex. As suggested by PCR data, precise HR in this plant formed the 5' recombination junction. However, the corresponding *Sca*I fragment in the gel blot analysis is larger than the correct 5' border junction fragment, suggesting the presence of additional sequences, either 5' to the recombination junction between the *Sca*I site and the Pubi_sense2 = binding site or in the 3' region of the *hpt* gene around the second *Sca*I site. In addition, the *Sma*I/*Hin*dIII fragment covering the 3' recombination junction including the *hpt* gene is also larger than predicted and the entire *pat* gene together with the adjacent BG212E29 right border sequences is deleted. The insertion in this plant (Fig. 5) might also have been generated by SDSA, initiated by the invasion of the left break end into a donor fragment and re-synthesis of the donor towards the right border. Thereby, template switching in the course of replication between the flanking *ubiquitin* promoter sequences, or HR between them, could have caused deletion of the entire *pat* gene. Alternatively, this deletion, induced by extrachromosomal HR, could have been present in the donor fragment already before strand invasion. Then, re-synthesis of the donor included the I-*Sce*I gene and continued up until close to the right border. Recombination at the right end then occurred by NHEJ via ligation to a largely resected right end of the break. Template switching between partially replicated molecules or replication slippage during re-synthesis could have produced the additional sequences of plant R1. Alternatively, recombination by crossover HR could have initiated integration at the 5' recombination junction and additional HR and/or NHEJ between the remaining target locus sequences, the additional rearranged recipient T-DNA copies that might be present in BG212E29, and the remaining donor sequences could have caused these rearrangements, including the elimination of the *pat* gene and the right border sequences.

The two heritable gene replacement events were obtained in one transformation experiment (UB53, Table 1) with DSB induction. Similar results are likely also to be obtained in further experiments, suggesting that high gene targeting efficiencies are achievable in barley when targeted DSB induction is applied. The targeted plants were obtained with an established standard procedure for transformation, limited labour input, and an output sufficient for practical application. The efficiency we obtained in barley is comparable with that observed previously by DSB induction with direct gene transfer (Shukla *et al.*, 2009; Townsend *et al.*, 2009; Ainley *et al.*, 2013) or *Agrobacterium*-mediated transformation using highly efficient nucleases (D’Halluin *et al.*, 2008; Cai *et al.*, 2009). Different nucleases such as ZFNs, TALEN, CRISPR–Cas, or meganucleases can differ greatly in their efficiency to induce a DSB. Quality and quantity of expression and the design of the artificial nuclease are major factors determining the efficiencies of DSB induction (Beumer *et al.*, 2013; Qi *et al.*, 2013; Gurushidze *et al.*, 2014; Johnson *et al.*, 2015; Mikami *et al.*, 2015). The meganuclease I-*Sce*I is one of the best DSB induction tools known (Puchta *et al.*, 1996; Puchta, 1998; Voytas, 2013). In addition, our version was optimized for barley and its DSB induction capacity validated (Vu *et al.*, 2014). These data suggest that the efficiency of the nuclease played an important role in obtaining a high gene targeting efficiency. Another factor could be the targeting strategy. While other concepts use recipients with stably transformed nuclease genes integrated in the genome (De Pater *et al.*, 2009), we and others (D’Halluin *et al.*, 2008; Cai *et al.*, 2009; Shukla *et al.*, 2009; Townsend *et al.*, 2009) co-transferred this gene together with the donor construct. This design avoids cutting the target sequence before the donor construct is available, and thus prevents premature inactivation of the site by NHEJ before it can be used for gene replacement.

In conclusion, our data show that a high efficiency of gene targeting, well within the range for routine application, is achievable in barley. Key to the success may be the choice of proper tools, such as co-transfer of a highly expressed nuclease gene together with the donor construct. In any case, the achieved high efficiency of gene targeting and the recent development of new generations of synthetic nucleases set the stage for precision genome engineering as a routine tool for barley.

## Supplementary data

Supplementary data are available at *JXB* online.

Figure S1. Relevant DNA sequences of plant R3.

Figure S2. Supplemental PCRs.

Figure S3. PCR analysis of PAT/p6U-pro candidate plants.

Supplementary Data
