# Supplementary material for: Stable gene replacement in barley by targeted double-strand break induction
Source: J Exp Bot. 2015 Dec 27;67(5):1433–45. doi: 10.1093/jxb/erv537 (PMC4762383; doi:10.1093/jxb/erv537)
Supplement: Supplementary Data [file supp_67_5_1433__index.html]

Stable gene replacement in barley by targeted double-strand break induction — Stable gene replacement in barley by targeted double-strand break induction — Supplementary Data 

# Stable gene replacement in barley by targeted double-strand break induction

## Supplementary Data

Data files

- supplementary\_figures\_S1\_S3.pdf - Supplementary Data
