# Supplementary material for: Stable gene replacement in barley by targeted double-strand break induction
Source: J Exp Bot. 2015 Dec 27;67(5):1433–45. doi: 10.1093/jxb/erv537 (PMC4762383; doi:10.1093/jxb/erv537)
Supplement: Supplementary Data [file supp_erv537_supplementary_figures_S1_S3.pdf]

Journal of Experimental Botany

Stable gene replacement in barley by targeted double-strand break induction.

Koichi Watanabe, Ulrike Breier, Götz Hensel, Jochen Kumlehn, Ingo Schubert and Bernd Reiss

Figure S1

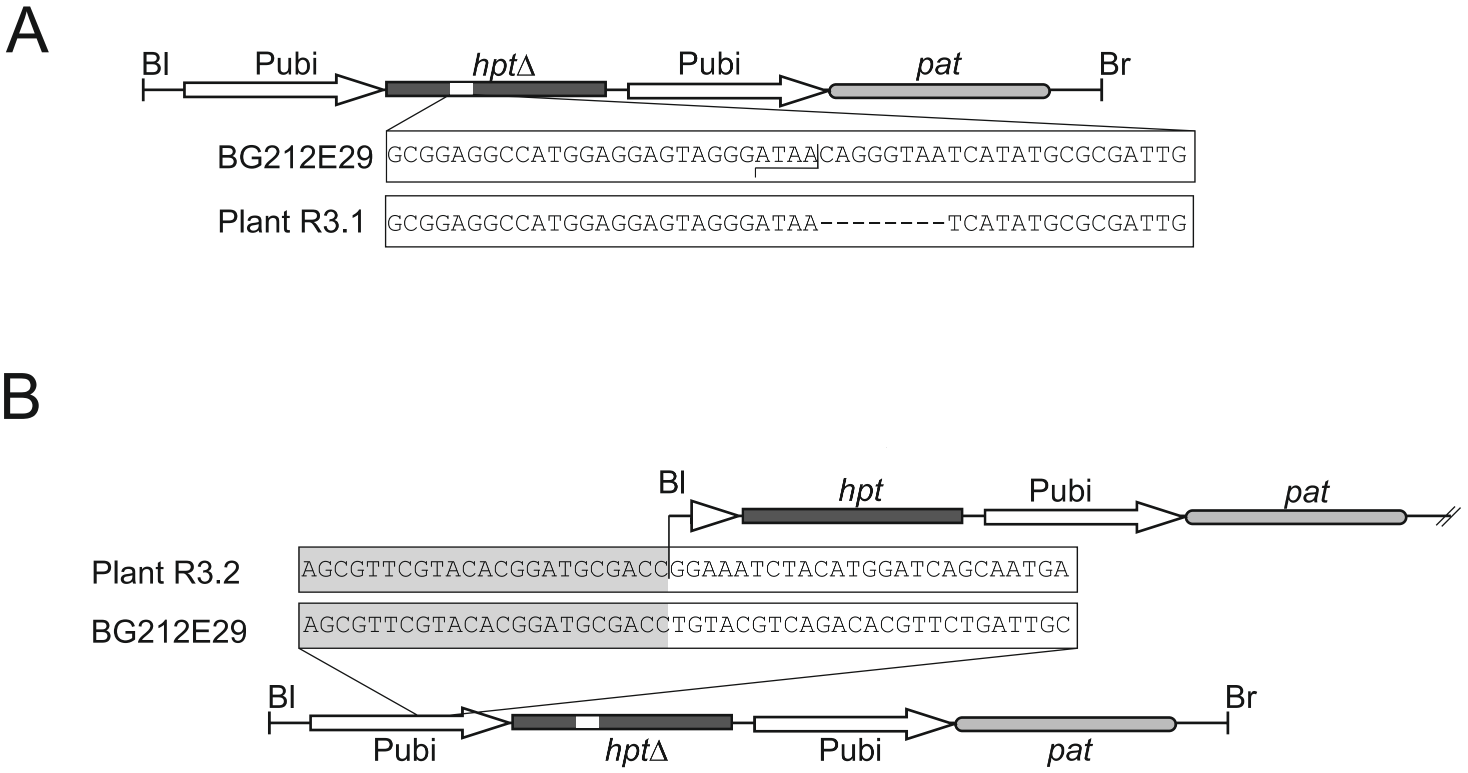

Figure S1: Relevant DNA sequences of plant R3. (**A**) A part of the I-*Sce*I site sequence in BG212E29 together with their genomic context is shown on top. The aligned sequence of plant R3.1 is shown below. The sequence shows a deletion of 8 bp in the I-*Sce*I recognition site directly next to the site of cleavage. (**B**) The 5' recombination junction of plant R3.2 is shown. The scheme shows the 5' region of the donor construct on top and the target locus at the bottom. The target locus sequence around the integration site is shown above. The aligned sequence above is the R3.2 DNA sequence. The part shaded in grey is identical to the BG212E29 sequence, while the white part in R3.2 is identical with the left border sequence of PAT-I-SceI/p6U-pro. The alignment shows the integration point of the T-DNA left border into the *ubiquitin* promoter in BG212E29. Symbols are as in Figure 1.

Figure S2

A

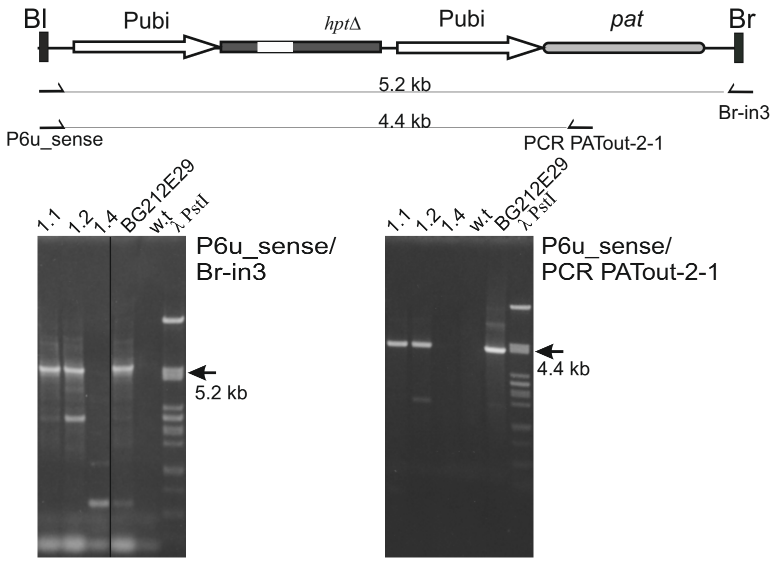

B

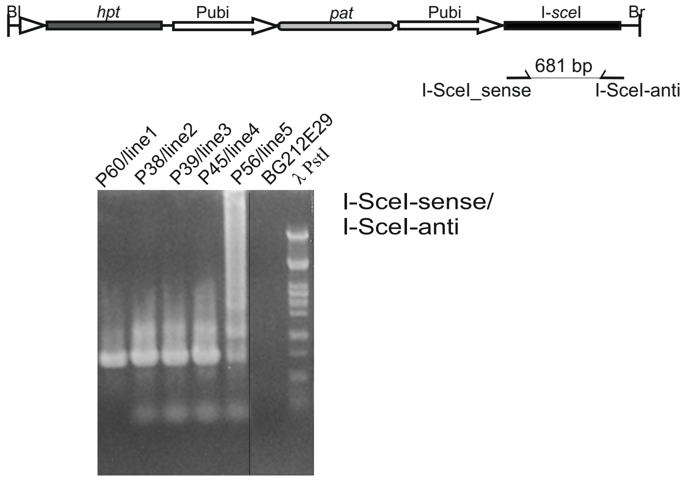

Figure S2: Supplemental PCRs. (**A**) The sequences in the 3' terminal part of the target locus are deleted in plant R1. The sketch shows the localisation of the primers used in the analysis together with the length of the predicted products. The symbols are as in Figure 1. Primer pair P6u_sense/Br-in3 amplifies the entire target locus while pair P6u_sense/pcrPATout-2-1 amplifies the portion until the *pat* gene. The results shown below indicate that the entire 3' part of the target locus including the *pat* coding region and the right border sequence has been deleted upon gene replacement. (**B**) The I-*Sce*I gene coding region is present in primary re-transformants. The scheme shows the donor construct on top together with the location of the primers used in the PCR analysis and the size of the expected product. The symbols are as in Figure 1. The products obtained from primary re-transformants R1 - R5 with primers I-*Sce*I-sense/ I-*Sce*I-anti are shown below. The results show that at least plants R1 - R4 have the I-*Sce*I gene stably integrated in the genome. The picture shows an EtBr stained agarose gel. The size standard shown on the right is *Pst*I digested phage λ DNA.

Figure S3

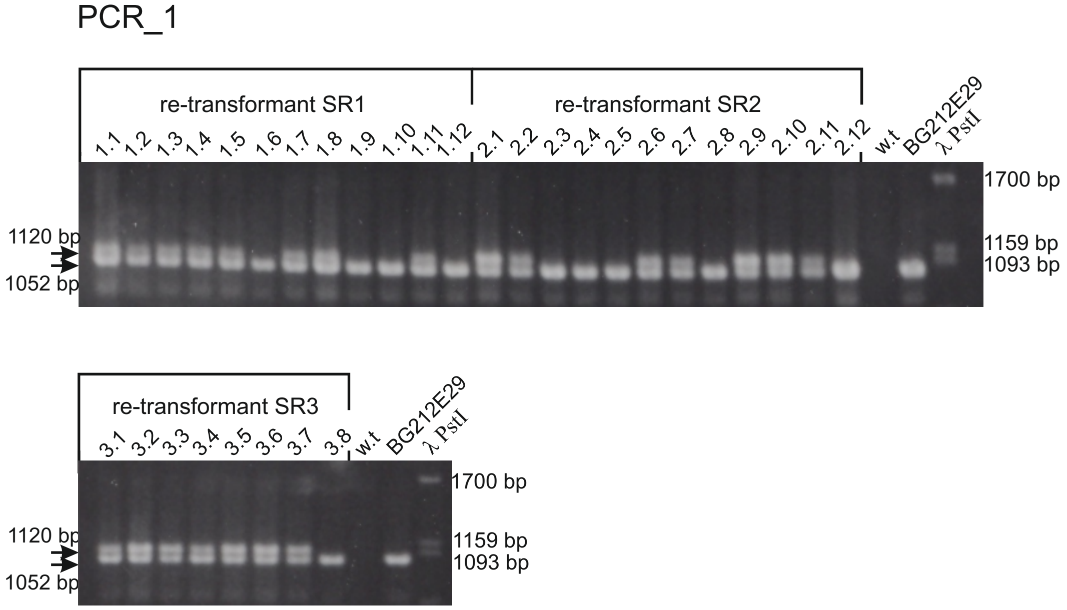

Figure S3: PCR analysis of PAT/p6U-pro candidate plants. Results of the segregation analysis of the progeny from 3 candidate plants obtained with PAT/p6U-pro. DNA of siblings in the progeny of plants SR1, SR2, and SR3 was prepared, amplified with PCR_1 and the products separated by high resolution (1.5%) agarose gel electrophoresis, the gels stained with EtBr and photographed. The analysis shows the presence of an unmodified target locus (1052 bp fragment) in all siblings of all 3 plants. An additional fragment representing the modified locus (1120 bp fragment) segregates independently on top of that in SR1 and SR2, and at least two such fragments in plant SR3. These results show that none of the SR1-SR3 plants were targeted. The fragment sizes in the visible part of the phage λ *Pst*I DNA length standard are given at the right end of the gel picture.
